# Association of Myocardial Enzyme Abnormality with Clinical Outcomes of Patients with COVID-19: A Retrospective Study

**DOI:** 10.1155/2021/3440714

**Published:** 2021-10-22

**Authors:** Qilin Zhang, Ziming Zheng, Yu Zhang

**Affiliations:** ^1^Department of Pharmacy, Union Hospital, Tongji Medical College, Huazhong University of Science and Technology, 430030 Wuhan, China; ^2^Hubei Province Clinical Research Center for Precision Medicine for Critical Illness, 430030 Wuhan, China

## Abstract

**Background:**

It has been observed that COVID-19 may cause myocardial damage, but there are few detailed reports on myocardial enzyme abnormalities.

**Methods:**

In this retrospective study, we analyzed data from 157 consecutive laboratory-confirmed and hospitalized COVID-19 patients from Wuhan. We collected information on demographic and clinical characteristics, laboratory findings, and clinical outcomes. Logistic regression analysis was used to explore the risk factors associated with the severity of COVID-19. The association between myocardial enzyme abnormalities and the mortality was also investigated.

**Results:**

The mortality in abnormal myocardial enzyme group was obviously higher than the normal group (*P* < 0.001). The majority of patients (*n* = 72, 97.3%) with normal cardiac enzyme group were of the common novel coronavirus pneumonia (NCP) type, whereas half of the patients with cardiac enzyme abnormalities (*n* = 40, 48.2%) developed critical and severe NCP type. The multivariable logistic regression analysis indicated that COVID-19 patients with increasing age (*P* = 0.035), higher levels of CRP (*P* = 0.038), and TNI (*P* = 0.036) were associated with increased death than other patients.

**Conclusions:**

Myocardial enzyme abnormality and myocardial injury were associated with the severity and fatal outcomes of COVID-19. Clinicians should pay attention to the markers of myocardial injury in COVID-19 patients, especially those with older age, comorbidities, and inflammation.

## 1. Introduction

Coronavirus disease 2019 (COVID-19) is still a big challenge for public health and medical care. Previous studies have reported the general clinical characteristics of hospitalized patients with COVID-19, including signs, symptoms, imaging features, laboratory findings, therapeutic strategies and effects, and multiple organ dysfunction [1, 2]. With the increase of confirmed cases and the accumulating clinical data, the cardiovascular manifestation caused by COVID-19 has attracted great attention. Huang et al. reported that 12% of patients had COVID-19-associated acute myocardial injury (AMI), manifested by elevated levels of high-sensitive troponin I ^1^. In a report on 138 patients with COVID-19, 16.7% had arrhythmias, and 7.2% had AMI [3]. The American College of Cardiology clinical bulletin has highlighted the cardiac implications of COVID-19 [4]. Thus, special medical attention should be paid to cardiac function-related indicators of COVID-19 patients. Myocardial enzymes are important indicators for judging myocardial function [5]. Even if there is no deliberate detection for myocardial enzymes, LDH and CK are often included in blood biochemical tests. However, to date, specific information characterizing whether patients with COVID-19 with myocardial enzyme abnormality face greater risk and have worse in-hospital outcomes remains unclear. The present study investigated the association of myocardial enzyme abnormality with fatal outcomes of patients with COVID-19.

## 2. Methods

### 2.1. Study Population

This study was approved by the institutional ethics board of the Union Hospital of Huazhong University of Science and Technology (Wuhan, China). Writing informed content was waived because it is a retrospective study. 157 consecutive patients with laboratory-confirmed COVID-19 admitted to Union Hospital of Huazhong University of Science and Technology between January 12, 2020, and February 26, 2020, were included in this retrospective cohort study. Patients with COVID-19 were diagnosed and classified as common or severe type according to the World Health Organization interim guidance. The cases without cardiac markers were excluded.

### 2.2. Data Collection

Data on demographic and clinical characteristics (age, sex, and symptoms), underlying comorbidities, laboratory findings, and cardiac markers, were collected from the electronic medical records. Laboratory test results including blood routine tests, coagulation profiles, blood glucose and lipids, inflammatory markers, and cytokine, liver, and renal function were recorded and compared. Cardiac markers measured on admission were collected, including cardiac troponin I (TNI), creatine kinase (CK), lactate dehydrogenase (LDH), and creatine kinase-myocardial band (CK-MB). Cardiac injury was defined as serum levels of TNI above the 99th-percentile upper reference limit (>26.2 *μ*g/L). All data were independently reviewed and entered into the computer database by 2 analysts (Q.Z. and Z.Z.). Patients were categorized according to the presence or absence of myocardial enzyme abnormality and myocardial injury. The clinical outcomes (i.e., discharges and mortality) were monitored up to February 26, 2020.

### 2.3. Statistical Analysis

Statistical analysis was performed using SPSS version 26.0 (Armonk, NY; GraphPad version 7.00, San Diego, CA). Continuous variables were presented as median (interquartile range (IQR)) and mean (standard deviation (SD)) for nonnormally distribution and normally distribution, respectively. Categorical variables were presented as *n* (%). Mann–Whitney *U* test or *χ*^2^ test was used to compare differences between presence and absence of myocardial enzyme abnormality or myocardial injury where appropriate. To explore the risk factors associated with myocardial enzyme abnormality and myocardial injury, univariable logistic regression analysis, Pearson's correlation analysis, and Spearman's rank correlation analysis were used for binary variables, normal distributed continuous variables, and nonnormal distributed continuous variables, respectively. To explore the risk factors associated with in-hospital death, univariable and multivariable logistic regression models were used. Considering the number of deaths (*n* = 18) in this study and to avoid collinearity between variables and overfitting in the model, five variables were selected for multivariable analysis based on previous findings and clinical constraints.

## 3. Results

### 3.1. Demographic and Clinical Characteristics

A total of 331 consecutive COVID-19 adult inpatients (18 years old) in the medical record system were screened initially from January 12, 2020, to February 26, 2020. Patients without available medical information and missing core results of laboratory examination (TNI, CK-MB, LDH, and CK) and duplicated records were excluded. Ultimately, 157 patients were included in the final analysis.

The demographic and clinical characteristics are summarized in Table 1. 83 (52.9%) patients were defined as the myocardial enzyme abnormality group as indicated by elevated TNI, CK, LDH, or CK-MB levels, and myocardial enzymes of 74 (47.1%) patients were within the normal range. Among these patients, fever (60 (72%)), cough (46 (57.3%)), and chest pain/tightness (29 (36.9%)) were the most common symptoms. On admission, 42 patients were critical or severe cases, and another 115 patients were diagnosed as nonsevere cases. Compared with patients with normal cardiac enzymes, those with abnormal cardiac enzymes were older (57 vs. 36 years; *P* < 0.001) and had a higher proportion of men (52 of 83 patients (62.7%) vs. 31 of 74 patients (41.9%); *P* = 0.009). Furthermore, patients with abnormal cardiac enzymes had significantly higher rates of comorbidities, including hypertension (27 (32.5%) vs. 11 (14.9%); *P* = 0.01), diabetes (15 (18.1%) vs. 2 (2.7%); *P* = 0.002), and chronic pulmonary disease (7 (8.4%) vs. 1 (1.4%); *P* = 0.044) (Table 1). Notably, patients with two or more than two comorbidities were more likely to show abnormalities in cardiac enzymes (19 (22.9%) vs. 5 (6.8%); *P* = 0.005). The proportion of critical and severe cases in the abnormal myocardial enzyme group was obviously higher than the normal group (40 (48.2%) vs. 2 (2.7%); *P* < 0.001).

### 3.2. Laboratory Findings

As shown in Table 1, compared with the normal group, patients in the abnormal group presented higher white blood count (median 6.13 vs. 4.91 10^9^/L), neutrophil count (median 4.6 vs. 2.74 10^9^/L), and lower lymphocyte count (median 1 vs. 1.43 10^9^/L) (all *P* < 0.001). The inflammatory markers, including CRP (median 31.87 vs. 4.55 mg/L), erythrocyte sedimentation rate (ESR) (median 51 vs. 13 mm/h), SAA (median 329.6 vs. 13.65 mg/L), and IL-6 (median 14.96 vs. 4.5 ng/L), were significantly higher in patients with abnormal cardiac enzymes (all *P* < 0.001). Besides, patients in the abnormal group also had higher levels of cardiac enzymes, such as LDH (median 353 vs. 179.5 U/L; *P* < 0.001), CK (median 99 vs. 46.5 U/L; *P* < 0.001), TNI (median 9.2 vs. 2.3 U/L; *P* < 0.001), and CK-MB (median 2.1 vs. 0.4 U/L; *P* = 0.016). As for liver and kidney function, those with abnormal cardiac enzymes also had higher levels of alanine aminotransferase (ALT) (median 36 vs. 21.5 U/L; *P* = 0.001), aspartate aminotransferase (AST) (median 33 vs. 22 U/L; *P* < 0.001), *γ*-transglutaminase (median 31 vs. 16 U/L; *P* < 0.001), and urea nitrogen (median 4.45 vs. 3.81 mmol/L; *P* = 0.011). The level of high-density lipoprotein cholesterol decreased more in abnormal group patients (median 0.9 vs. 1.05 mmol/L; *P* = 0.036). However, there is no significant difference in creatinine, uric acid, total cholesterol, triglyceride, and low-density lipoprotein cholesterol between the two groups.

### 3.3. Clinical Outcomes

**Figure 1:**
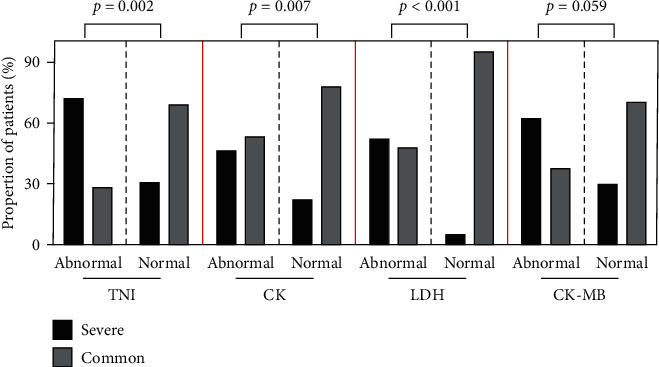
The proportions of severe COVID-19 patients in different cardiac enzyme groups.

In our study, a total of 18 patients (11.5%) died, and the rest (139 (88.5%)) were recovered and discharged as shown in Table 1. The mortality of patients with abnormal myocardial enzymes was significantly higher than patients in the normal group (18 (21.7%) vs. 0 (0%); *P* < 0.001). Moreover, nearly half of the patients with cardiac enzymes abnormalities (*n* = 40, 48.2%) developed critical and severe novel coronavirus pneumonia (NCP) type. The proportion of patients with elevated TNI, CK, or LDH levels who developed critical and severe NCP type was markedly higher than that of normal levels, with ratios of 72.2% vs. 31% (*P* = 0.002), 46.7% vs. 22.2% (*P* = 0.007), and 52.1% vs. 4.8% (*P* < 0.001), respectively (Figure 1).

### 3.4. Mortality of Patients with COVID-19 with/without Elevated TNI Levels

As shown in Table 2, the mortality rate of patients with myocardial injury was higher than those without myocardial injury (11 of 18 patients (61.1%) vs. 4 of 58 patients (6.9%); *P* < 0.001). Patients with cardiac injury had older age (66 years old (IQR 56.5, 78) vs. 48 years old (IQR 35, 64); *P* < 0.001) and were more likely to develop critical and severe COVID-19 cases (72.2% vs. 31%, *P* = 0.002). As shown in Table 3, the univariable logistic regression analysis demonstrated that the mortality rate increased in association with older age (*P* < 0.001), comorbidities (*P* = 0.003), respiratory rate (*P* < 0.001), white blood cells (*P* < 0.001), neutrophils (*P* < 0.001), platelets (*P* < 0.001), levels of TNI (*P* = 0.014), CK (*P* = 0.003), and LDH (*P* < 0.001), and inflammatory markers, which including CRP (*P* < 0.001), procalcitonin (*P* = 0.017), ESR (*P* = 0.017), SAA (*P* = 0.034), and IL-6 (*P* = 0.004), and indicators of liver function, which including ALP (*P* = 0.004), AST (*P* = 0.009), total bilirubin (*P* = 0.030), and direct bilirubin (*P* = 0.049), and levels of urea nitrogen (*P* < 0.001). Furthermore, from the multivariable logistic regression analysis, we found that COVID-19 patients with increasing age (OR, 1.143; 95CI% (1.009, 1.295); *P* = 0.035), higher levels of CRP (OR, 1.026; 95CI% (1.001, 1.052); *P* = 0.038), and TNI (OR, 1.018; 95CI% (1.001, 1.035); *P* = 0.036) were associated with increased death than other patients (Table 3).

## 4. Discussion

Previous study has found that the novel coronavirus may attack many important organs [6]. Heart and myocardial injury might be involved and even be regarded as one of the leading causes of death of COVID-19 patients [7]. However, few studies concentrated on the effect of COVID-19 infection on the heart, and the degree of myocardial damage on the clinical outcome and prognosis remained unclear. Therefore, our study performed a retrospective analysis to report the association between cardiac abnormalities including elevated myocardial enzyme levels (52.87%) and myocardial injury (11.46%) and fatal outcomes of patients with COVID-19.

According to a recent study of Wuhan city, severe cases of COVID-19 were independently associated with older age, elevated levels of hs-TNI, CK-MB, and myohemoglobin [4]. It was notable that a previous report in 138 COVID-19 patients found that 7.2% of patients had cardiac injury [3], which was confirmed by another report in 187 patients with COVID-19 indicating 27.8% of patients exhibited myocardial injury as indicated by elevation of cardiac troponin T levels [8]. Besides, in 2003, a study from Guangzhou institute of respiratory diseases showed that CK-MB and LDH increased in 10.8% and 16.0% of patients with severe acute respiratory syndrome (SARS), respectively [9]. These observations suggested that myocardial injury and abnormal cardiac enzymes played greater roles in the fatal outcomes of COVID-19 patients. In our current study, of the 157 patients with COVID-19, 83 showed abnormal cardiac enzymes, and 18 exhibited myocardial injury. In abnormal cardiac enzyme group, both the mortality rate and proportion of severe cases were higher than the normal group. Compared with patients without myocardial injury, patients with myocardial injury presented with more severe NCP type (72.2% vs. 31%), more cardiovascular disease (CVD, 33.3% vs. 13.8%), and higher mortality rate (61.1% vs. 6.9%), manifested by abnormal laboratory findings, such as older age, higher levels of CRP, ESR, SAA, IL-6, TNI, AST, ALP, and urea nitrogen, demonstrating that cardiac injury in COVID-19 patients was associated with major adverse clinical outcomes.

Cardiac complications, including abnormal cardiac enzymes, cardiac injury, heart failure, and arrhythmias, were common in patients with pneumonia. Risk factors of cardiac events after pneumonia usually include older age, coexisting cardiovascular diseases, inflammatory, and severity of NCP type. Similarly, Chen reported that in critical and severe patients with COVID-19, the markers of myocardial injury and inflammation including TNI, N-terminal probrain natriuretic peptide (NT-proBNP), and high-sensitivity CRP were significantly elevated and associated with mortality [10]. Increased age, even male sex, hypertension, and coronary heart disease were additional risk factors leading to severity of pneumonia. Consistently, our study indicated that older age, coexisting diseases, especially for CVD, inflammatory, TNI levels, and function of the liver and kidney were possible risk factors of COVID-19 patients, which were also in associated with myocardial injury.

Previously study believed that increased age was an important independent factor of mortality in MERS and SARS [11, 12]. Our study also identified that older age was associated with cardiac injury and death of COVID-19 patients. Because of elderly patients with poor immunity, especially the lower function of T lymphocytes and multiple comorbidities, the inflammatory cytokines such as IL-6 and TNF-*α* would be abnormally elevated contributing to cytokine storms, and they were more likely to develop into severe pneumonia potentially leading to poor outcome when infected with virus as compared to young patients.

In addition, large studies reported that CVD was a risk factor for COVID-19 patients [13, 14]. A study of 1099 COVID-19 patients demonstrated that the proportion of patients had coexisting disease reached to 23.7% and hypertension (15%), diabetes (7.4%), and coronary heart disease (2.5%) were the top three underlying diseases [13]. Supporting this point of view, our current study indicated that patients with underlying CVD and other comorbidities were more likely to develop abnormal cardiac enzymes, elevated TNI levels, and more severe NCP type.

It is notable that a recent report on 112 patients hospitalized with COVID-19 who have experienced a definite outcome found that most of patients had normal levels of TNI at the beginning of infection while 37.5% patients increased during hospitalization, especially in those with severe clinical conditions [15]. In a similar study of 138 patients, 36 patients required ICU admission, and their levels of CK-MB and hs-TNI were significantly higher than the non-ICU group [3]. Further, previous study also reported that levels of cardiac troponin and myoglobin in the severe and death group were prominently elevated than the mild and discharge group [16, 17]. Particularly, in our present study, 23.68% of patients showed elevated TNI levels and also had a markedly odd ratio of 1.018 (95% CI (1.001-1.035)) for the risk of myocardial injury and death triggered by COVID-19. Moreover, other indicators of cardiac injury including CK and LDH were also independent risk factors of COVID-19 death. Thus, it was rational to resume that the elevation in cardiac TNI could be a warning sign for the death of patients with COVID-19 which should be evaluated and paid more attention to in clinical practice.

The potential mechanism of COVID-19-induced myocardial injury was not well known so far. Some researchers believed that inflammatory response might be at least partially responsible for cardiac injury. A recent report on 416 patients found that markers of inflammatory response, such as CRP, procalcitonin, and leukocytes, were significantly increased among patients with cardiac injury [4]. Chen et al. also reported that patients with COVID-19 who suffered from more severe pneumonia had higher levels of IL-6 and other inflammatory cytokines [10]. In our current study, we found that high levels of myocardial injury markers were significantly in association with increase of white blood cells and neutrophils and decrease of lymphocytes in patients with abnormal cardiac enzymes, which were consistent with other COVID-19 studies. Moreover, other inflammatory cytokines including CRP, IL-6, ESR, and SAA were also elevated. The release of these inflammatory cytokines could lead to myocardial inflammatory cell infiltration and inflammatory storm, which would contribute to increase of myocardial cell apoptosis and necrosis. Supporting our point of view, in a retrospective study of 463 cases of COVID-19 patients, researchers found that severe and aged COVID-19 patients suffered more obvious decreases in total lymphocytes, CD4^+^, and CD8^+^ T lymphocytes, suggesting SARS-CoV-2 infection might affect heart by inflammation and cytokine storm [18]. Collectively, we speculated that severe inflammatory response might be a possible mediator of myocardial injury caused by COVID-19. Therefore, early and special anti-inflammatory treatment should be undertaken in clinical.

Another mechanism for COVID-19 caused myocardial injury might be associated with angiotensin converting enzyme 2 (ACE2). Studies reported that ACE2 had strong binding affinity to the spike protein of SARS-CoV-2 and could contribute to myocardial inflammation and damage [19]. SARS-CoV virus RNA was detected in human heart samples, indicating SARS-CoV-2 might directly attack heart by binding ACE2 on myocardial cells [20]. The expression of ACE2 protein in heart samples was markedly downregulated by invasion of SARS-CoV-2 virus, which not only caused impaired endothelial function, elevated blood pressure, and reduced myocardial contraction force but also increased the angiotensin II. Finally, increased oxidative stress, inflammation, and fibrosis in heart were occurred by activating TGF-*β* and MAPK pathways [21]. So far, the use of ACEI/ARB in COVID-19 patients remained controversy. A large study of 1178 cases in Wuhan suggested that ACEI/ARB were not associated with the severity or mortality of COVID-19 patients with hypertension [22]. Therefore, we might conclude that for COVID-19 patients who previously used ACEI/ARB, the use of these drugs might not need to be discontinued based on the current data.

Our study had several limitations. Firstly, only 157 patients with confirmed COVID-19 were included, and the number of patients with elevated TNI levels was limited. Therefore, a larger cohort was needed to verity our conclusions. Secondly, some information about myocardial injury, such as echocardiography, electrocardiogram, and NT-proBNP levels, was not presented in the study because the data was incomplete partly due to the limited conditions of extraction. Thirdly, the mechanism of potential myocardial injury and inflammation caused by COVID-19 was not specific, which required more evidence from the long-term observation and prospective study design. Our present findings might provide clinicians with some evidence to pay attention to the cardiac pathology in future especially for patients with elevated cardiac enzyme levels on admission.

## 5. Conclusions

Myocardial injury was associated with the severity and fatal outcomes of COVID-19. SARS-CoV-2 virus might attack heart by inducing inflammatory storm or binding with ACE2. Clinicians should pay attention to the markers of myocardial injury in COVID-19 patients, especially those with older age, comorbidities, and inflammation. Finally, early and special anti-inflammatory or myocardial protection treatment should be undertaken for patients with COVID-19 in clinical.

## Figures and Tables

**Table 1:** Demographic, clinical, and laboratory findings of patients on admission.

	Normal range	Total (*n* = 157)	Cardiac enzyme abnormality (*n* = 83)	Without cardiac enzyme abnormality (*n* = 74)	*Z*/*χ*2	*P* value
*Demographics and clinical characteristics*						
Age, y		48 (34-64)	57 (43-67)	36 (31-48.5)	-5.708	<0.001
Sex (men)		83 (52.9%)	52 (62.7%)	31 (41.9%)	6.766	0.009
Fever		113 (72.0%)	60 (72.3%)	53 (71.6%)	0.009	0.926
Cough		90 (57.3%)	46 (55.4%)	44 (59.5%)	0.261	0.61
Chest pain/tightness		58 (36.9%)	29 (34.9%)	29 (39.2%)	0.303	0.582
Respiratory rate		20.7 (2.8)	21.3 (3.7)	20.0 (0.8)		0.004
Systolic pressure	90-140 mm Hg	126.5 (117-138)	131.5 (120-140)	122.5 (115-130)	-3.046	0.002
Diastolic pressure	60-90 mm Hg	78 (72-88.75)	79 (70-90)	78 (74-87)	-0.027	0.978
*Comorbidities*						
Hypertension		38 (24.2%)	27 (32.5%)	11 (14.9%)	6.655	0.01
Diabetes		17 (10.8%)	15 (18.1%)	2 (2.7%)	9.571	0.002
Other CVD		22 (14.0%)	13 (15.7%)	9 (12.2%)	0.398	0.528
CPD		8 (5.1%)	7 (8.4%)	1 (1.4%)	4.058	0.044
With more than two comorbidities		23 (14.6%)	19 (22.9%)	5 (6.8%)	7.865	0.005
*Laboratory findings*						
Blood routine tests						
White blood cell	3.5-9.5 10^9^/L	5.31 (4.32-7.11)	6.13 (4.52-8.73)	4.91 (3.9-6.1)	-3.59	<0.001
Neutrophil	1.8-6.3 10^9^/L	5.25 (3.3-8.3)	4.6 (2.87-7.46)	2.74 (1.99-3.77)	-5.282	<0.001
Lymphocyte	1.1-3.2 10^9^/L	1.23 (0.88-1.64)	1 (0.61-1.31)	1.43 (1.13-1.82)	-5.25	<0.001
Platelet count	125-350 10^9^/L	196 (154-246)	196 (153.5-260)	198 (153.5-236)	-0.188	0.851
Haemoglobin	130-175 g/L	125 (115-138)	121 (110-138)	126 (117-139)	-1.62	0.105
Monocyte count	0.1-0.6 10^9^/L	0.37 (0.27-0.54)	0.36 (0.26-0.55)	0.39 (0.29-0.53)	-0.799	0.424
*Blood glucose and lipids*						
FBG	4.1-5.9 mmol/L	4.99 (4.58-6.1)	5.75 (4.82-6.47)	4.93 (4.49-5.26)	-3.185	0.001
Total cholesterol^∗^	<5.2 mmol/L	3.95 (0.88)	3.82 (1.05)	4.05 (0.73)		0.22
Triglyceride	<1.7 mmol/L	1.21 (0.9-1.52)	1.2 (0.93-1.76)	1.23 (0.89-1.47)	-0.591	0.555
HDL	1.16-1.42 mmol/L	0.99 (0.8-1.27)	0.9 (0.71-1.21)	1.05 (0.85-1.29)	-2.091	0.036
LDL^∗^	2.7-3.1 mmol/L	2.33 (0.69)	2.22 (0.83)	2.41 (0.57)		0.18
*Inflammatory markers and cytokines*						
CRP	<8 mg/L	10.8 (3.39-35.78)	31.87 (8-77.83)	4.55 (3.14-11.38)	-6.588	<0.001
Procalcitonin	<0.5 *μ*g/L	0.13 (0.13-0.20)	0.15 (0.13-0.49)	0.13 (0.07-0.15)	-5.242	<0.001
SAA	<10 mg/L	37.7 (6.6-403.95)	329.6 (28.7-674.8)	13.65 (3.93-32.83)	-5.326	<0.001
ESR	<20 mm/h	19 (9-35.5)	51 (23-64)	13 (6.75-22.25)	-5.598	<0.001
IL-6	0.12-2.9 ng/L	6.92 (3.54-18.39)	14.96 (6.35-29.18)	4.5 (3.02-9.17)	-4.912	<0.001
IL-10	0.1-5 ng/L	3.83 (3.09-5.24)	5.05 (3.64-6.23)	3.45 (2.9-4.29)	-4.832	<0.001
Cardiac markers						
CK	138-174 U/L	62 (41.25-120.75)	99 (52-222.25)	46.5 (33.75-67)	-5.743	<0.001
LDH	109-245 U/L	233 (180.5-357)	353 (268-510)	179.5 (160.7-203.2)	-9.962	<0.001
TNI	<26.2 *μ*g/L	4.8 (1.2-18)	9.2 (4.7-119.4)	2.3 (0.3-4.2)	-5.207	<0.001
CK-MB	0-24 *μ*g/L	1.5 (0.325-15)	2.1 (0.65-15.5)	0.4 (0.2-15)	-2.404	0.016
Liver and renal function						
ALT	5-40 U/L	26 (17.5-48.5)	36 (20.5-57)	21.5 (15-41.75)	-3.466	0.001
AST	8-40 U/L	26 (20-41)	33 (25-56.5)	22 (17.25-27.75)	-5.522	<0.001
ALP	40-150 U/L	63 (50-80)	66 (50.5-88)	56 (50-72.5)	-2.122	0.034
*γ*-Transglutaminase	11-50 U/L	23 (14-43.5)	31 (18.5-57.5)	16 (12-33.5)	-4.053	<0.001
Total bilirubin	5.1-19 *μ*mol/L	10.25 (8-13.78)	12.05 (8.6-15.3)	9.15 (7.38-11.68)	-3.307	0.001
Urea nitrogen	2.9-8.2 mmol/L	4 (3.19-5.57)	4.45 (3.19-8.31)	3.81 (3.18-4.76)	-2.557	0.011
Creatinine	46-92 *μ*mol/L	68.2 (58.05-81.6)	69.55 (58.13-90.9)	66.3 (57.3-76.8)	-1.754	0.079
Uric acid	149-369 *μ*mol/L	264.7 (200.7-335.9)	256.4 (195.03-335.48)	281.1 (208.3-345.2)	-1.055	0.292
*Clinical typing and outcome*						
Severe		42 (26.8%)	40 (48.2%)	2 (2.7%)	41.314	<0.001
Nonsevere		115 (73.2%)	43 (51.8%)	72 (97.3%)		
Death		18 (11.5%)	18 (21.7%)	0		<0.001
Discharge		139 (88.5%)	65 (78.3%)	74 (100%)		

CVD: cardiovascular and cerebrovascular diseases; CPD: chronic pulmonary disease; FBG: fasting blood glucose; HDL: high-density lipoprotein; LDL: low-density lipoprotein; CRP: C-reactive protein; SAA: serum amyloid A; ESR: erythrocyte sedimentation rate; IL-6: interleukin-6; IL-10: interleukin-10; CK: creatine kinase; LDH: lactate dehydrogenase; TNI: cardiac troponin I; CK-MB: creatine kinase-myocardial band; ALT: alanine aminotransferase; AST: aspartate aminotransferase; ALP: alkaline phosphatase. ^∗^Continuous variables were presented as mean (standard deviation (SD)). Other continuous variables were presented as median (interquartile range (IQR)).

**Table 2:** Demographic and clinical characteristics of patients with/without myocardial injury on admission.

	Total (*n* = 76)	With myocardial injury (*n* = 18)	Without myocardial injury (*n* = 58)	*Z*/*χ*2	*P* value
Age, median, (range), y	54 (36-66)	66 (56.5-78)	48 (35-64)	-3.502	<0.001
Sex (men)	44 (57.9%)	12 (66.7%)	32 (55.2%)	0.745	0.388
Fever	54 (71.1%)	9 (50.0%)	45 (77.6%)	5.083	0.024
Cough	42 (55.3%)	9 (50.0%)	33 (56.9%)	0.264	0.607
Chest pain/tightness	32 (42.1%)	7 (38.9%)	25 (43.1%)	0.1	0.752
Respiratory rate^∗^	21 (3.39)	22.3 (4.36)	20.7 (3.02)		0.09
Systolic pressure, mm Hg	128 (117-138)	124 (101-140)	130 (118.75-138)	-1.517	0.129
Diastolic pressure, mm Hg	80 (73-89)	75 (65.5-90)	80 (76-89)	-1.646	0.1
Hypertension	25 (32.9%)	6 (33.3%)	19 (32.8%)	0.002	0.964
Diabetes	11 (14.5%)	3 (16.7%)	8 (13.8%)	0.092	0.762
Cardiovascular and cerebrovascular diseases	15 (19.7%)	7 (38.9%)	8 (13.8%)	5.461	0.019
Chronic pulmonary disease	6 (7.9%)	4 (22.2%)	2 (3.4%)	6.658	0.01
With more than two comorbidities	15 (19.7%)	5 (27.8%)	10 (17.2%)	0.963	0.327
Severe	31 (40.8%)	13 (72.2%)	18 (31.0%)	9.649	0.002
Common	45 (73.2%)	5 (27.8%)	40 (69.0%)		
Death	15 (19.7%)	11 (61.1%)	4 (6.9%)	25.487	<0.001
Discharge	59 (77.6%)	6 (33.3%)	53 (91.4%)		

^∗^Continuous variables were presented as mean (standard deviation (SD)). Other continuous variables were presented as median (interquartile range (IQR)).

**Table 3:** Risk factors associated with in-hospital death.

	Univariable OR (95% CI)	*P* value	Multivariable OR (95% CI)	*P* value
*Clinical characteristics*				
Age, years^∗^	1.128 (1.07-1.189)	<0.001	1.143 (1.009-1.295)	0.035
Female sex (vs. male)	0.618 (0.23-1.664)	0.341	1.091 (0.108-11.007)	0.941
Respiratory rate^∗^	1.325 (1.138-1.542)	<0.001	—	—
Chest pain/tightness#	1.28 (0.483-3.392)	0.620	—	—
Underlying comorbidities#	4.787 (1.705-13.438)	0.003	0.561 (0.035-9.02)	0.683
*Laboratory findings*				
Platelets^∗^	0.977 (0.966-0.988)	<0.001	—	—
Neutrophils^∗^	1.415 (1.207-1.658)	<0.001	—	—
WBC^∗^	1.315 (1.142-1.514)	<0.001	—	—
IL-6^∗^	1.018 (1.006-1.031)	0.004	—	—
IL-10^∗^	1.229 (0.991-1.524)	0.060	—	
CRP^∗^	1.031 (1.018-1.045)	<0.001	1.026 (1.001-1.052)	0.038
SAA^∗^	1.003 (1-1.005)	0.034	—	—
ESR^∗^	1.031 (1.006-1.057)	0.017	—	—
Procalcitonin^∗^	1.654 (1.093-2.504)	0.017	—	—
ALP^∗^	1.014 (1.004-1.023)	0.004	—	—
ALT^∗^	0.998 (0.987-1.009)	0.683	—	—
AST^∗^	1.007 (1.002-1.013)	0.009	—	—
*γ*-Transglutaminase^∗^	1.002 (0.995-1.01)	0.500	—	—
Total bilirubin^∗^	1.062 (1.006-1.121)	0.030	—	—
Direct bilirubin^∗^	1.069 (1-1.142)	0.049	—	—
Total bile acid^∗^	1.066 (0.976-1.165)	0.158	—	—
Urea nitrogen^∗^	1.714 (1.339-2.149)	<0.001	—	—
Creatinine^∗^	1.004 (0.999-1.009)	0.085	—	—
EGFR^∗^	0.952 (0.932-0.972)	<0.001	—	—
CK^∗^	1.002 (1.001-1.004)	0.003	—	—
LDH^∗^	1.004 (1.002-1.007)	<0.001	—	—
TNI^∗^	1.009 (1.002-1.017)	0.014	1.018 (1.001-1.035)	0.036
*Disease severity*				
Severe vs. common	2.621 (1.342-5.117)	0.005	—	—

OR: odds ratio; WBC: white blood cell; IL-6: interleukin-6; IL-10: interleukin-10; CRP: C-reactive protein; SAA: serum amyloid A; ESR: erythrocyte sedimentation rate; ALP: alkaline phosphatase; ALT: alanine aminotransferase; AST: aspartate aminotransferase; EGFR: glomerular filtration rate; CK: creatine kinase; LDH: lactate dehydrogenase; TNI: cardiac troponin I. ^∗^Per 1 unit increase. #Present vs. not present.

## Data Availability

The data used to support the findings of this study are available from the corresponding author upon request.

## Supplementary Materials

Supplementary 1 Table S1: risk factors associated with myocardial enzyme abnormality.

Supplementary 2 Table S2: risk factors associated with acute myocardial injury.
