# Supplementary material for: Association of Myocardial Enzyme Abnormality with Clinical Outcomes of Patients with COVID-19: A Retrospective Study
Source: Dis Markers. 2021 Oct 22;2021:3440714. doi: 10.1155/2021/3440714 (PMC8556588; doi:10.1155/2021/3440714)
Supplement: Supplementary 1 — Table S1: risk factors associated with myocardial enzyme abnormality. [file 3440714.f1.docx]

Table S1 Risk factors associated with myocardial enzyme abnormality

|  | Univariable OR  (95% CI) and r/r_s_ | *P* value |
| --- | --- | --- |
| Clinical characteristics | | |
| Age, years§ | -0.457 | <.001 |
| Female sex ( v.s. male) | 0.43 (0.226-0.816) | .010 |
| Fever# | 1.034 (.515-2.076) | .926 |
| Cough# | 0.848 (0.449-1.599) | .610 |
| Respiratory rate§ | -0.235 | .004 |
| Chest pain/tightness# | 0.833 (0.435-1.595) | .582 |
| Underlying comorbidities# | 3.435 (1.72-6.86) | <.001 |
| Laboratory findings | | |
| Platelets* | -0.27 | .738 |
| Monocytes§ | 0.064 | .426 |
| Neutrophils§ | -0.426 | <.001 |
| WBC§ | -0.289 | <.001 |
| IL-6§ | -0.458 | <.001 |
| IL-10§ | -0.451 | <.001 |
| CRP§ | -0.547 | <.001 |
| SAA§ | -0.568 | <.001 |
| ESR* | -0.610 | <.001 |
| ALP§ | -0.174 | .033 |
| ALT§ | -0.285 | <.001 |
| AST§ | -0.454 | <.001 |
| γ-transglutaminase§ | -0.333 | <.001 |
| Total bile acid§ | -0.273 | .001 |
| Urea nitrogen§ | -0.215 | .010 |
| Creatinine§ | -0.144 | .079 |
| Uric acid* | 0.038 | .648 |
| EGFR§ | 0.281 | <.001 |
| **Disease severity** | | |
| Severe v.s. Non-severe | 33.488 (7.704-145.577) | <.001 |

OR=odds ratio. WBC=white blood cell. IL-6=interleukin-6. IL-10=interleukin-10. CRP=C-reactive protein. SAA=serum amyloid A. ESR=erythrocyte sedimentation rate. ALP=alkaline phosphatase. ALT=alanine aminotransferase. AST=aspartate aminotransferase. EGFR=glomerular filtration rate. *Pearson's correlation coefficient (r) for continuous variables with normal distribution. §Spearman's rank correlation coefficient (r_s_) for continuous variables with non-normal distribution. # Present v.s. Not-present for binary variables.
