# Supplementary material for: Association of Myocardial Enzyme Abnormality with Clinical Outcomes of Patients with COVID-19: A Retrospective Study
Source: Dis Markers. 2021 Oct 22;2021:3440714. doi: 10.1155/2021/3440714 (PMC8556588; doi:10.1155/2021/3440714)
Supplement: Supplementary 2 — Table S2: risk factors associated with acute myocardial injury. [file 3440714.f2.docx]

Table S2 Risk factors associated with acute myocardial injury

|  | Univariable OR (95% CI) and r/r_s_ | *P* value | |
| --- | --- | --- | --- |
| Clinical characteristics | | | |
| Age, years§ | -0.404 | <.001 | |
| Female sex ( v.s. male) | 0.615 (0.203-1.864) | .391 | |
| Fever# | 0.261 (0.085-0.801) | .019 | |
| Cough# | 0.758 (0.262-2.187) | .608 | |
| Respiratory rate§ | -0.161 | .175 | |
| Chest pain/tightness# | 0.840 (0.285-2.475) | .752 | |
| Underlying comorbidities# | 4.62 (1.355-15.754) | .014 | |
| Laboratory findings | | |  |
| Platelets* | 0.175 | .133 | |
| Monocytes§ | 0.361 | .001 | |
| Neutrophils§ | -0.310 | .007 | |
| WBC§ | -0.216 | .062 | |
| IL-6§ | -0.203 | .131 | |
| IL-10§ | -0.193 | .149 | |
| CRP§ | -0.465 | <.001 | |
| SAA§ | 0.029 | .867 | |
| ESR* | -0.014 | .927 | |
| ALP§ | -0.391 | .001 | |
| ALT§ | -0.100 | .409 | |
| AST§ | -0.382 | .001 | |
| γ-transglutaminase§ | -0.202 | .090 | |
| Total bile acid§ | -0.271 | .023 | |
| Urea nitrogen§ | -0.470 | <.001 | |
| Creatinine§ | -0.102 | .396 | |
| Uric acid* | -0.009 | .942 | |
| EGFR§ | 0.293 | .010 | |
| CK§ | -0.284 | .013 | |
| LDH§ | -0.464 | <.001 | |
| CK-MB§ | -0.557 | <.001 | |
| **Disease severity** | | |  |
| Severe v.s. Non-severe | 2.621 (1.342-5.117) | .005 | |

OR=odds ratio. RBC=red blood cell. WBC=white blood cell. IL-6=interleukin-6. IL-10=interleukin-10. CRP=C-reactive protein. SAA=serum amyloid A. ESR=erythrocyte sedimentation rate. ALP=alkaline phosphatase. ALT=alanine aminotransferase. AST=aspartate aminotransferase. EGFR=glomerular filtration rate. CK=creatine kinase. LDH=lactate dehydrogenase. CK-MB=creatine kinase-myocardial band. *Pearson's correlation coefficient (r) for continuous variables with normal distribution. §Spearman's rank correlation coefficient (r_s_) for continuous variables with non-normal distribution. # Present v.s. Not-present for binary variables.
